# Pooled DNA genotyping on Affymetrix SNP genotyping arrays

**DOI:** 10.1186/1471-2164-7-27

**Published:** 2006-02-15

**Authors:** George Kirov, Ivan Nikolov, Lyudmila Georgieva, Valentina Moskvina, Michael J Owen, Michael C O'Donovan

**Affiliations:** 1 Department of Psychological Medicine, Henry Wellcome Building, Cardiff University, Heath Park, Cardiff CF14 4XN, UK

## Abstract

**Background:**

Genotyping technology has advanced such that genome-wide association studies of complex diseases based upon dense marker maps are now technically feasible. However, the cost of such projects remains high. Pooled DNA genotyping offers the possibility of applying the same technologies at a fraction of the cost, and there is some evidence that certain ultra-high throughput platforms also perform with an acceptable accuracy. However, thus far, this conclusion is based upon published data concerning only a small number of SNPs.

**Results:**

In the current study we prepared DNA pools from the parents and from the offspring of 30 parent-child trios that have been extensively genotyped by the HapMap project. We analysed the two pools with Affymetrix 10 K Xba 142 2.0 Arrays. The availability of the HapMap data allowed us to validate the performance of 6843 SNPs for which we had both complete individual and pooled genotyping data. Pooled analyses averaged over 5–6 microarrays resulted in highly reproducible results. Moreover, the accuracy of estimating differences in allele frequency between pools using this ultra-high throughput system was comparable with previous reports of pooling based upon lower throughput platforms, with an average error for the predicted allelic frequencies differences between the two pools of 1.37% and with 95% of SNPs showing an error of < 3.2%.

**Conclusion:**

Genotyping thousands of SNPs with DNA pooling using Affymetrix microarrays produces highly accurate results and can be used for genome-wide association studies.

## Background

Single nucleotide polymorphisms (SNPs) are the most abundant type of polymorphism in the human genome. With the parallel developments of dense SNP marker maps and technologies for high-throughput SNP genotyping, SNPs have become the markers of choice for genetic association studies. The use of dense but incomplete maps of SNP markers for genetic association is based upon the premise that low penetrance but fairly common disease variants can be detected by virtue of indirect association between SNP markers and disease status. As a general rule, the denser the map of markers used, the greater the probability that at least one marker will be in strong linkage disequilibrium (LD) with a disease susceptibility allele, and therefore indirect association between marker and disease will be detected [1].

With the development of genotyping platforms that permit analysis of several hundreds of thousands of markers, it is now possible to apply this principle of indirect association to the whole genome rather than just candidate genes or candidate linkage regions. For example Affymetrix (Santa Clara, California), recently released microarrays that can interrogate ~500,000 SNPs, and Illumina (San Diego, California) released in January 2006 the Sentrix(r) HumanHap300 Genotyping BeadChip which can genotype 317,504 high-value SNP loci derived principally from tag SNPs. Theoretical predictions [2] as well as empirical data concerning the structure and distribution of LD in the human genome [3] suggest that analyses on this scale will probably be adequate for whole genome association studies targeted at common disease variants.

The number of subjects required to detect the influence of a risk allele by indirect association depends upon the locus-specific genotype relative risks conferred by the susceptibility variant and the maximum LD between it and any assayed marker. For unknown loci, these parameters can only be guessed, but the expectation is that the relative risks will usually be small and therefore the required samples large. Substantial samples are also required to offset the enormous degree of multiple testing inherent in genome-wide studies. Thus an uncorrected threshold for statistical significance of α = 10^-7 ^is required to achieve a genome-wide type I error rate of only 0.05 in the face of testing 500,000 independent SNPs. Although this is somewhat conservative since many markers are in LD (and therefore the tests are not independent), it serves as a rough approximation to the scale of the statistical burden. These dual considerations of small genetic effect sizes and adjustment for multiple testing have led many to assume that samples in the region of at least 1000 or more cases and a similar number of controls will be required for most complex disorders [e.g. [4-6]]. Given these expected sample sizes, while genome-wide association are indeed technically feasible, they are also expensive.

One way to reduce the cost is to undertake quantitative analyses of allele frequencies in DNA pools, a process often referred to as 'DNA pooling' [7,8]. Here, equal amounts of DNA from patients and controls are mixed to form two sets of pools. The pools are then genotyped and the frequency of each allele estimated. The power of such studies is approximately the same as for individual genotyping of cases and controls [4,9], but at a hugely reduced cost. DNA pooling has proved remarkably accurate when applied to simple tandem repeats [10-13] or to SNPs using a variety of different genotyping technologies [7]. Typically, when estimates of allele frequency differences between two pools are compared with those obtained by individual genotyping, the mean error rate of pooled analysis is in the region of 1–2%.

Several groups have begun to apply pooled genotyping to the new ultra-high throughput genotyping technologies. Butcher et al, 2004 [14] and Meaburn et al, [15] pioneered this method by assessing the performance of the Affymetrix 10 K Array Xba 131 for pooled genotyping. They validated by individual genotyping pooling data obtained from 10 SNPs in their first experiment [14] and 104 SNPs in the follow-up work [15]. They also compared the pooled data from the remaining markers on the chip with allele frequency data from a reference Caucasian population. The same group recently [16] reported an applied DNA pooling study based upon the 10 K Array with mild mental impairment as a phenotype. They followed up the pooling data for the 12 most significant markers by individual genotyping in a larger replication sample. Four of these SNPs remained significantly associated. Liu et al, [17] recently reported the results of a study where pools of 20 individuals each were used to identify differences between substance abusers and controls (a total of 1253 individuals were genotyped). This strategy allowed them to identify 38 "nominally reproducibly positive" SNPs.

Although these studies give cause for optimism, it is clear that the validity of pooled genotyping using array technology has not been proven for a sufficiently large number of SNPs to allow researchers to apply the method with confidence. In this paper, we have undertaken a more comprehensive analysis of the accuracy of microarray-based pooling experiments. Rather than examine a small selection of SNPs, we examined 6843 fully informative SNPs out of a total of 10,204 SNPs represented on the Affymetrix 10 K Xba 142 2.0 array. Our results suggest that pooled genotyping using Affymetrix arrays is as accurate as that obtained with lower throughput platforms, and that it can be performed instead of individual genotyping with only a minimal loss of power.

## Results

### Reproducibility

**Figure 1:**
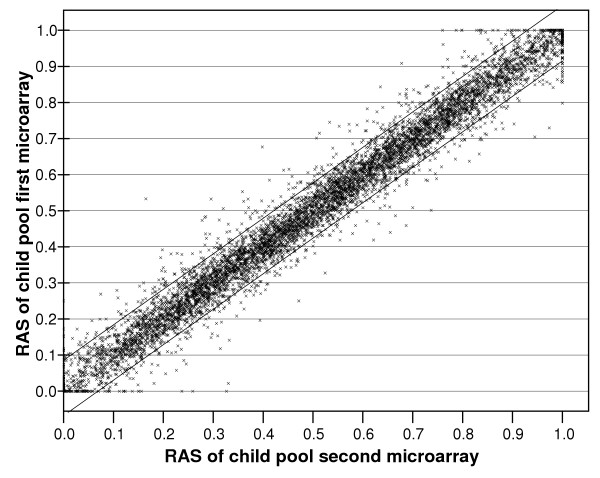
Reproducibility of RAS values for the sense strands obtained for the same pool on two independent arrays. The two flanking lines capture 95% of the data points. The correlation coefficient is *r *= 0.985.

To estimate allele frequencies in pooled DNA samples, we used the Relative Allele Signal (RAS) scores given in the output of the Affymetrix GeneChip DNA Analysis Software (GDAS). RAS scores are produced separately for the sense and antisense strand for each SNP and can be analysed separately, or can be averaged. The test-retest estimates of allele frequency in pools were high for duplicate experiments. This is illustrated in Figure 1 in which estimates of allele frequency at 6843 SNP loci obtained in one array (for the sense strand only) are compared with the same data from another single array. When all possible pairs of such data were analysed, the mean correlation between single arrays ranged from *r *= 0.974 to 0.986. The correlations between sense only analyses and between antisense only analyses were virtually identical.

**Figure 2:**
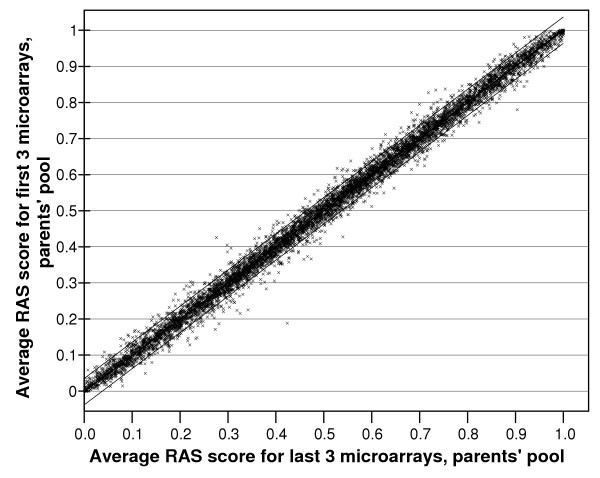
Averaged RAS values from sense and antisense strands obtained from 3 microarrays, compared with the data of the same pool of DNA from an independent set of 3 microarrays. The lines capture 95% of the data points. The correlation coefficient is *r *= 0.996.

While the average correlations are strong for any pair of arrays, the spread of the data with respect to individual SNPs, as depicted by the width of the bounded zone capturing 95% of the data points (Figure 1), clearly shows weak reproducibility for a large number of individual markers. We therefore attempted to reduce measurement errors by using the repeated measures of the same pool. When the RAS scores for the sense and anti-sense strands in a single array were averaged, reproducibility improved, with mean correlations now ranging between *r *= 0.985–0.992. The correlation continued to improve when data from replicate arrays were included. With a maximum of 6 arrays performed on a single (parental) sample, our data allow us to compare the composite data from 3 arrays with what should be identical data from an independent set of the other 3 arrays. As each array has sense and antisense data, we have a total of 6 observations per pool. Even at this fairly modest degree of replication, very high reproducibility was obtained, with an *r *= 0.996. Equally important, the bounded zone containing 95% of the data is much narrower (Figure 2).

### Allele frequency estimation

**Figure 3:**
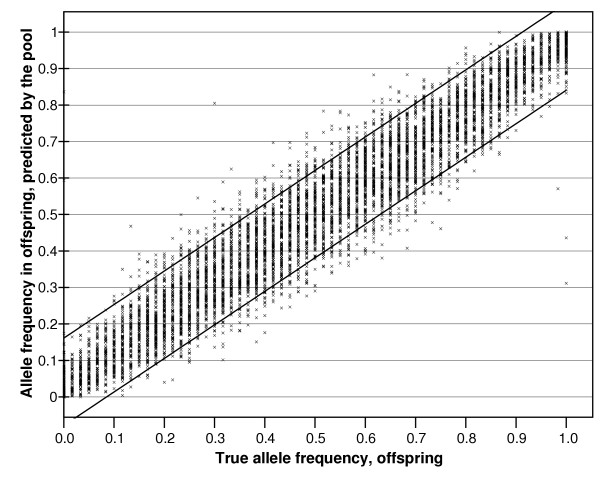
Allele frequencies in offspring (uncorrected with *k *for bias in allele representation) predicted by pooled analysis (y-axis) plotted against the true allele frequencies obtained by individual genotyping of all subjects from whom the pool was derived (x-axis). The correlation coefficient is *r *= 0.961. True frequency differences are plotted in intervals of 0.0083 which corresponds to increments of one allele in the 30 offspring.

We averaged the RAS values (combining sense and antisense strands) from the five replicate measures of the offspring pool and the six replicate measures of the parental pool. The true allele frequencies in the parental and the offspring samples were calculated from the HapMap genotype database. Without correction with *k *for unequal representation of alleles (see Methods) the allele frequencies we estimated from the pooled analyses correlated well with the true frequencies derived for each sample from the HapMap (*r *= 0.959 for the parents and 0.961 for the offspring). The data for the offspring sample are shown in Figure 3.

**Figure 4:**
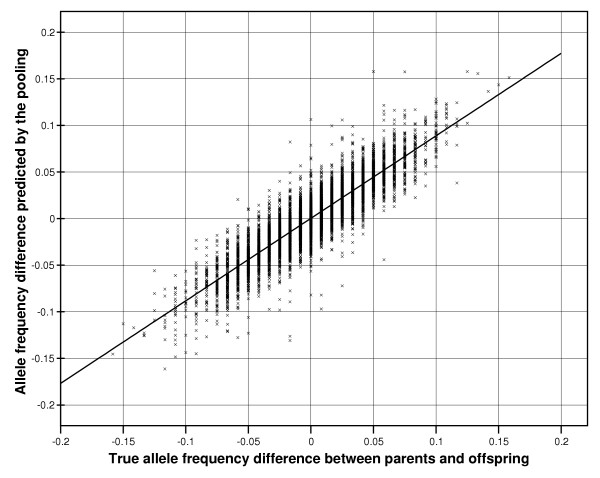
Correlation between the true allele frequency differences between parents and offspring (as determined by individual genotyping) and the allele frequency differences estimated from pooled analyses.

**Figure 5:**
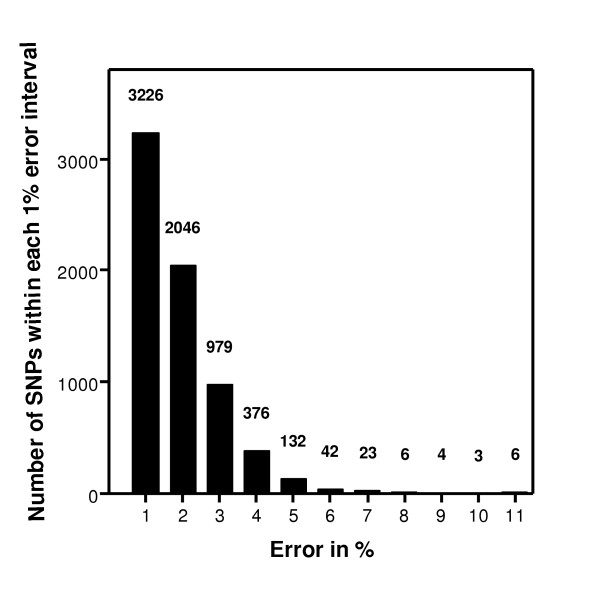
Bar chart depicting the distribution of errors arising from pooled analyses of allele frequency differences between parents and offspring. The x-axis shows the size of the error in intervals of 1% and the y-axis shows the number of SNPs falling within each interval. The number of SNPs within each 1% interval are also given. The mean error = 1.37%, range 0–11.4%. 95% of SNPs have an error of ≤ 3.2%.

While the correlation is high, the spread of the data does not allow confidence that any single allele frequency can be accurately predicted. However the main aim of pooled analysis is to predict differences in frequencies between pools rather than the absolute allele frequencies *per se*. The true allele frequency differences between parents and offspring were calculated from the HapMap data and compared with the allele frequency differences predicted from the pooled analyses. The results (uncorrected with *k *for unequal allele representation, see Methods) are presented in Figure 4. The mean error in estimating the allele frequency differences between the two pools was only 1.37%, with 95% of all results showing an error of <3.2% and 99% of results <4.6%. The error distribution in bins of 1% is shown in Figure 5.

**Figure 6:**
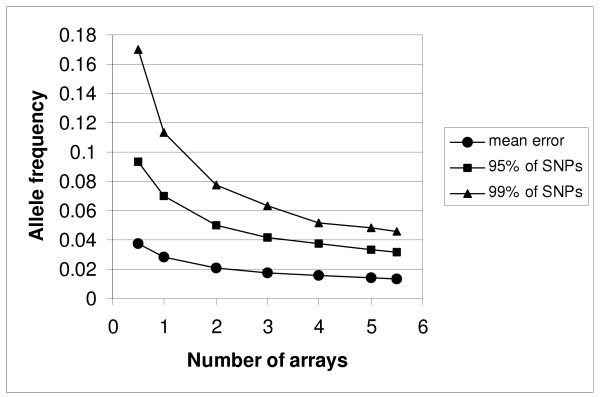
Change in error (y-axis) arising from pooled analyses of allele frequency differences between parents and offspring with increasing number of arrays. The x-axis shows the number of arrays used for each sample. At position 0.5 we show the data for a single (sense or antisense) strand. The final observation (position 5.5) is based upon 5 arrays for children and 6 arrays for parents (mean 5.5). Mean errors are represented by circles, and error thresholds below which 95% (squares) and 99% (triangles) of the data lie.

We estimated how the distribution of the error in pooled analysis varies with attempts to reduce measurement error through repeat measurement. The results are presented in Figure 6 which shows how the mean error in estimating allele frequency differences between pools changes with increasing number of replicate analyses. We also present the error thresholds below which lie 95% and the 99% of the data. The number of arrays used per pool is plotted on the x axis. The x point of 5.5 corresponds to analysis of 5 arrays for offspring and 6 for parents (a mean of 5.5 arrays per sample). The x data-point of 0.5 corresponds to the data obtained for just one strand (sense or antisense) for each array (i.e. half the data captured by each array). With each replicate, we obtain an improvement in the mean error and a lowering of the thresholds for 95% and 99% of the data. Although in each case the degree of improvement begins to plateau, even the addition of the final replicate contributes to reduction in error.

### Correcting the data

**Figure 7:**
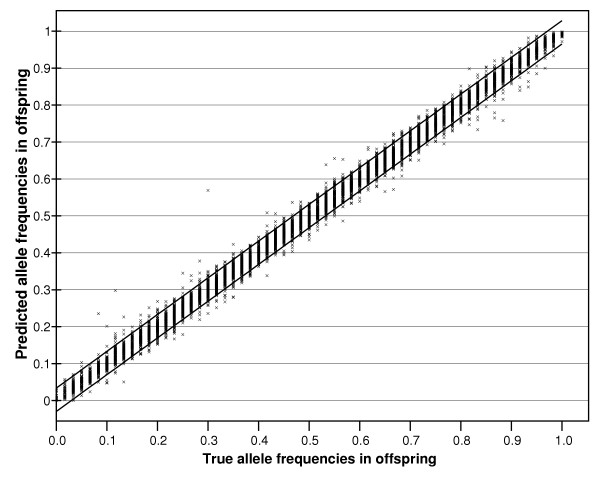
Allele frequencies in the offspring pool predicted by pooled analysis and corrected with *k *(y-axis), plotted against the true allele frequencies of this sample (x-axis). The correlation coefficient is *r *= 0.997. Note that the coefficient *k *was derived from parental data (see methods) and then applied to the data obtained for children. Any errors in estimating the parental allele frequencies are incorporated in the estimate of *k *and therefore the estimate of the allele frequency in offspring includes the measurement errors from both samples.

We performed several sets of corrections to see if our results can be improved. First we applied *k*, a correction factor often used in pooling experiments to correct the data for any unequal efficiencies in representing alleles [18] (see Methods section). As for other pooling methods [7], this procedure improves the correlation between measured and true allele frequencies (*r *= 0.997) and also reduces the spread of the data (compare Figure 7 with Figure 3). For a small proportion of SNPs, the true allele frequency in the pools was substantially different from the measured allele frequency in that pool. Where this occurs, extreme values of *k *are derived (see Methods) for the use in other pools. For technical reasons which are entirely understandable, this usually occurs for low frequency SNPs. Consider a putative SNP that is actually non-polymorphic (allele frequency = 1 or 0). RAS scores are seldom exactly zero. The result is that *k *reaches 0, (or alternatively 8) depending upon which allele is arbitrarily designated allele A (see Methods). Since the use of inappropriate values for *k *markedly affects the type I and type II errors where either the true allele frequencies are very low and/or the correct *k *values extreme [18], the data for SNPs whose derived *k *is small or large may not be reliable.

We therefore discarded 286 SNPs whose values of *k *were >5 or <0.25: (including 100 SNPs where *k *= 0 and 110 SNPs where *k *= ∞) and re-examined the distribution of errors. This procedure had little impact on the error in estimating differences between pools, with no change in the mean error (data not shown). This is not surprising since this procedure affected only SNPs with very small minor allele frequencies (mostly non-polymorphic SNPs) which cannot reach big differences between the pools used in our experiment. Correction with *k *is likely however to a have a substantial effect in true case-control association studies, where a small difference in the frequencies of rare alleles can easily result in significant p-values. (In the current experiment we decided not to analyse p-values due to the very small sample size of the pools involved.)

We also sought to identify poorly performing SNPs based upon large test-retest variation between arrays. Within each pool, we measured the difference between the highest and lowest RAS value for each SNP produced by the replicate arrays. We term this difference the "maximum variability of the results". Just over 5% of SNPs showed a maximum variability of >25% between the lowest and highest signal of any array. Given that SNPs which show a higher degree of variability are likely to produce less reliable results, we removed these 341 SNPs. This correction did not change substantially the error in estimating differences between pools.

## Discussion

We have investigated the accuracy and reproducibility of DNA pooling using the Affymetrix 10 K Xba 142 2.0 array by comparing pooled data with individual genotype data. In this respect, our analyses were greatly facilitated by the availability of individual genotype data for 6843 SNPs on all the individuals from whom we made pools, courtesy of the HapMap project. Our data show that pooled estimation of allele frequencies was highly reproducible. When we compared the allele frequencies calculated from the average of only three microarray replicates from one pool with that calculated from the average of a second set of three estimates for the same pool, the correlation between measures was *r *= 0.996 (Figure 2). When we use as our outcome measure the magnitude of the error in estimating differences in allele frequencies between samples (Figure 6), while continued improvement is evident with increasing number of arrays, our data show further improvements are rather small beyond 4 replicates per sample. Our data therefore suggest this as the minimum number of arrays that should be hybridised per pool, although more replicates are likely to further reduce the rate of false-positive and false-negative results.

Pooled analysis yielded accurate estimates of the *relative *allele frequencies in pools but not estimation of the *absolute *allele frequencies when the data were not corrected for unequal allele representation. Using the average values obtained from five (offspring) and six (parent) arrays as artificial case and control pools, the system performed well, with a mean error of just 1.37% which is comparable with other commonly used lower throughput methods [7]. 95% of all estimated differences between pools were within < 3.2%, and 99% of them were within < 4.6% of the true differences.

### Data correction

There are many reasons why alleles might be unequally represented even when present in equimolar amounts [8,19]. For microarrays, this might include differential allele specific PCR hybridisation efficiencies. Correction for this with a coefficient *k *has been shown to improve both type I and type II errors in DNA pooling experiments [8,18,19]. This is because the estimation of the statistical significance of any observed difference requires that the approximate true allele frequency in the sample be known, a process significantly improved by use of the coefficient *k*. The improvement in estimating true allele frequency is in fact better than is apparent from Figure 7. This is because *k *is derived from parental data (see methods) and then applied to the data obtained for children (or vice versa). Therefore any errors in estimating the parental allele frequencies are incorporated in the estimate of *k *with the result that the estimates of allele frequencies in offspring include the measurement errors from both samples.

Where the application of *k *is required, the solution until now has been to derive *k *from the ratio of the intensities (or whatever other measures are appropriate) of the signals representing each allele specific products from heterozygous individuals. To this aim, Simpson et al, [19] have recently initiated the development of a central resource for the accumulation of microarray data from heterozygous individuals. However, finding several heterozygous individuals for rare SNPs is problematic and the whole process rather complex if 500,000 SNPs need to be examined. Our approach avoids this problem. Instead of analysing heterozygous individuals, we suggest using HapMap reference samples (in our case CEPH trios) thus eliminating the need to obtain genotypes from heterozygotes for every SNP.

Under the (unproven) assumption that to some extent, the value of *k *varies with laboratory practice, the greatest accuracy will be achieved if each group constructs their own pools of CEPH individuals (or pools from reference panels from other ethnic groups if they are working with other ethnic groups) and then conducts their own array replicates (10 K, 100 K or 250 K) to derive their own laboratory specific *k *values as described in the Methods section. It should be noted that *k *corrects for technical artefacts in allele representation rather than population specific differences in allele frequency and that the *k *values derived in one sample are applicable to any other sample regardless of differences in allele frequency. However, the use of the most similar ethnic group represented in the HapMap to those comprising the main focus of research within a lab does have the advantage that *k *values for any population-specific SNPs can be derived (some SNPs are non-polymorphic in some populations).

However, it may well be the case that with the use of the highly standardised operating procedures which are always used for the work with Affymetrix arrays, inter-laboratory *k *values will be modest and in most cases insufficient to be a significant source of error [14,18]. This is an issue we are currently exploring and if confirmed, we will deposit *k *values for the two 250 K arrays on our institutions website, and/or provide them to researchers upon request.

In terms of practical utility, our results indicate that moderate differences in allele frequency should be easily detected by pooled analysis followed by a very limited amount of individual genotyping, thereby greatly enhancing the cost-efficiency of the study. As an example, let us assume we are interested in detecting an unknown SNP which has a frequency of 30% in the 1000 controls and 38% in 1000 cases. This scenario corresponds to an allele with an odds ratio of 1.4 and an association which surpasses our approximate genome-wide threshold for significance of p = 10^-7 ^(by analysis of a 2 × 2 contingency table). In our sample, 276 SNPs have true allele frequency differences >8% between parents and offspring. If we set as our target to individually genotype all SNPs which in pools showed an 8% difference, we would end up genotyping 286 SNPs which include 54% of the 276 SNPs with true 8% allele frequency differences (and all 10 SNPs with a frequency difference >13%). Thus, by undertaking the pooled experiment, we would have identified 54% of the target loci (frequency difference 8%) and all 10 best loci, but at the cost of genotyping only a very small proportion of the SNPs.

If we use the correction with *k*, our correct discovery rate remained similar at 50% (we would have discovered correctly 153 SNPs by genotyping 306 SNPs). However, most designs aim to follow up the results surpassing a given threshold of statistical significance. For a given sample size, the calculated statistical significance depends not just on the magnitude of the allele frequency difference between samples but also on the allele frequency. Our data concerning corrected and uncorrected data (compare Figures 3 and 7) clearly show that estimates of absolute allele frequencies are greatly improved by correcting for *k*. This correction improved the estimation of allele frequencies in the current study from a correlation with the true data of *r *= 0.961 for the offspring pool (Figure 3) to *r *= 0.997 when *k *correction was applied to that pool (Figure 7). Therefore we expect that when the best p-values in an experiment are targeted, then a correction with *k *will lead to an improvement of the discovery rate. Fortunately, with the method we propose, and the availability of genotyped reference samples, the process of deriving *k *is now quite straightforward.

We have illustrated the efficiency gains obtained by DNA pooling with Affymetrix arrays by choosing differences of 8% or more, but clearly this is a very arbitrary threshold and smaller differences are likely to be of interest to some researchers, particularly in larger samples. Useful cost efficiency gains can still be made, though self evidently, the smaller the difference sought, the less the absolute magnitude of the gains. Where the goal is to detect more modest differences in allele frequency, it is possible that cost-efficiency might be improved by more replicates. This is because even though our data show that the improvements in the mean error rate beyond 4 replicates are relatively small (Figure 6), the absolute number of SNPs falsely predicted by pooling continues to go down with more replicates.

### 10 K versus 250 K arrays

We have to consider whether the conclusions we have drawn with respect to the 10 K array are likely to be valid for the 250 K arrays (two of which when combined constitute the Affymetrix 500 K arrays). Each SNP is interrogated by 40 features on the 10 K array but by only 24 features on the 250 K array (a reduction from 10 to 6 quartets per SNP, although a small proportion of SNPs are represented on more quartets on the 250 K arrays). This reduction in the number of features per SNP, as well as the reduction of the feature size from 8 to 5 microns, may reduce information content. This suggests that more replicate arrays will be needed to achieve accuracy and reproducibility equivalent to that reported in the present study. A slight problem is created by the fact that for the 250 K arrays the Affymetrix software does not calculate automatically RAS scores. However, these can easily be calculated from the intensity values reported for each array feature, using the algorithms described by Liu et al, [20].

So far there have been only a few published fairly high density genome-wide association studies and these have so far been based on around 100,000 SNPs. Klein et al, [21] genotyped 116,204 SNPs in a sample of 96 patients with age-related macular degeneration, and 50 controls. They identified a SNP in the complement factor H gene (CFH) which was strongly associated with disease (nominal p value <10^-7^), a result that has been replicated since. Yamazaki et al, [22] genotyped 72,738 SNPs in 94 Japanese patients suffering with Crohn's Disease, and 752 controls. This lead to the discovery of a highly significant association with the TNFSF15 gene, p = 1.71 × 10^-14 ^in a large replication sample. Several more studies on a variety of phenotypes have been performed but have not been fully published yet. Summaries of such studies on multiple sclerosis and cardiovascular disease using 100,000 SNP, and Graft-Versus-Host disease using 500,000 SNPs are available in the Affymetrix Microarray Bulletin of April 2005 [23]. While the results of each study give cause to believe that genome association approaches can deliver novel genes, the costs are likely to prevent similar approaches being applied in all reasonably sized samples across the whole spectrum of complex disorders. Until the costs reduce further, our data suggest that as an interim step, DNA pooling based upon arrays will allow laboratories with relatively modest budgets to usefully contribute to this research.

## Conclusion

When performed on Affymetrix SNP genotyping arrays, DNA pooling provides a fairly accurate method for identifying allele frequency differences between samples, with a mean error of 1.37%. In order to minimise measurement error, the final data should be based upon composite scores from 4–5 replicates for 10 K arrays, but this number will probably need to be increased for 500 K arrays, or where smaller differences in allele frequency are required. While not essential for measuring differences in allele frequencies between samples, to obtain estimates of the statistical significance of those differences, the data should be corrected for unequal representation of the two alleles using the coefficient *k*. Instead of deriving *k *from the ratio of signals in heterozygous individuals, we suggest deriving it from pooling data obtained from samples of reference individuals from whom DNA and vast quantities of genome-wide genotype data are available through cell repositories and the HapMap respectively. The cost-efficiency of DNA pooling on arrays should enable even small laboratories to contribute to genome-wide association studies.

## Methods

We obtained DNA samples from 30 anonymous CEPH trios (90 individuals) from The Human Genetic Cell Repository at the Coriell Institute for Medical Research [24]. These are the trios that have been genotyped by the HapMap consortium. DNA pools were prepared using serial dilutions of the stock DNA and measuring the concentration after each dilution with the PicoGreen ds DNA Quantitation Reagent (Molecular Probes, Eugene, Oregon) on a Labsystems Ascent Fluoroscan (Life Sciences International, Basingstoke, UK). We aimed at a target concentration of approximately 50 ng/μl. From this working dilution we took equimolar amounts of DNA from each individual and prepared one pool from all 60 parents and another pool representing all 30 offspring. The final concentration of each pool was 46 ng/μl.

Genotyping with the Affymertix 10 K Xba 142 2.0 Array was performed at the MRC Rosalind Franklin Centre for Genomics Research, Wellcome Trust Genome Campus, Cambridge, UK using the standard protocol recommended by the manufacturer, with no special modification. Briefly, 250 ng of genomic DNA per array were digested with a restriction enzyme (Xba I) and ligated to adapters that recognise the cohesive four base-pair overhangs. A generic primer that recognises the adapter sequence was then used to amplify the adapter-ligated fragments in a PCR reaction. The amplified DNA was then fragmented, labelled and hybridised to the array. The Affymetrix GeneChip Operating Software (GCOS) collected and extracted feature data from the scanner, which was then analysed with the Affymetrix GeneChip DNA Analysis Software (GDAS). We performed 5 replicates (5 arrays) of the offspring pool and 6 replicates of the parent pool. The researcher performing the hybridisation and scanning of the chips was not informed of the details of our experiment.

### Data analysis

Each Affymetrix 10 K array includes more than 420,000 features, each consisting of over one million copies of a 25 bp oligonucleotide probe of a defined sequence synthesized by photolithographic manufacturing [25]. Each SNP is interrogated by five probe quartets for each strand of the DNA. Each quartet is composed of four probes of a perfect match and mismatch for alleles A and B of that SNP, giving a total of 40 different 25 bp nucleotides for each SNP, with each probe having a variation in perfect matches, mismatches and flanking sequence around the SNP. The software provides raw data for the intensity of fluorescence corresponding to the amount of labelled PCR product hybridised to each feature on the microarray. To convert these data to estimated allele frequencies for each SNP we used the median of the relative allele signals (RAS) of the 5 quartets that are available for each SNP on each strand. These RAS values are produced by the GDAS programme according to algorithms described by Liu et al, [20]. This procedure is the same used previously by Butcher et al, [14]. Although the software used for analysis of 250 K arrays does not produce RAS scores, these can be calculated using the same algorithms.

To estimate the accuracy of the pooled analysis, we compared our estimations with the true allele frequency differences between parents and offspring based upon the genotypic data available from HapMap. From the 10204 SNPs represented on the array, we first excluded 172 with no given chromosomal location, followed by 1780 SNPs that had not been genotyped by the HapMap project at the time we completed our analysis. We then excluded 1242 SNPs which had one or more individuals with missing HapMap genotypes because given the small sample sizes of our pools, data from even a single individual in the case of children contributes 0.033 to the estimated allele frequency, which is above the margin of error that we were hoping to achieve. Finally we excluded the remaining 167 SNPs on the X-chromosome because with respect to this chromosome, males and females do not contribute equimolar amounts of DNA to the pools. This left 6843 fully informative SNPs suitable for analysis.

### Estimation of the correction coefficient (k)

In pooling experiments it is generally customary to normalise the data by accounting for inequalities in the representation of each allele [18,26,27]. Such a procedure has also been reported as necessary for pooled analyses on microarrays [15]. The correction coefficient (*k*) is usually estimated as

*k *= *h*_*A*_/*h*_*B *_    (1)

where *h*_*A *_and *h*_*B *_are the measurements representing alleles A and B in heterozygous individuals, for example peak heights or signal intensities. This ratio is then used to normalize the data from pools in order to calculate *f*_*A *_(the predicted frequency of allele A):

fA=HAHA+kHB     (2)
 MathType@MTEF@5@5@+=feaafiart1ev1aaatCvAUfKttLearuWrP9MDH5MBPbIqV92AaeXatLxBI9gBaebbnrfifHhDYfgasaacH8akY=wiFfYdH8Gipec8Eeeu0xXdbba9frFj0=OqFfea0dXdd9vqai=hGuQ8kuc9pgc9s8qqaq=dirpe0xb9q8qiLsFr0=vr0=vr0dc8meaabaqaciaacaGaaeqabaqabeGadaaakeaacqWGMbGzdaWgaaWcbaGaemyqaeeabeaakiabg2da9maalaaabaGaemisaG0aaSbaaSqaaiabdgeabbqabaaakeaacqWGibasdaWgaaWcbaGaemyqaeeabeaakiabgUcaRiabdUgaRjabdIeainaaBaaaleaacqWGcbGqaeqaaaaakiaaxMaacaWLjaWaaeWaaeaacqaIYaGmaiaawIcacaGLPaaaaaa@3D68@

where *H*_*A *_and *H*_*B *_are the intensities of allele A and B in the pool. In the current experiment we know the exact allele frequency for each SNP from the HapMap data, therefore we can estimate the correction coefficient without the need to observe heterozygous individuals. For this purpose we first express *k *from formula (2):

k=HA(1−fA)fAHB
 MathType@MTEF@5@5@+=feaafiart1ev1aaatCvAUfKttLearuWrP9MDH5MBPbIqV92AaeXatLxBI9gBaebbnrfifHhDYfgasaacH8akY=wiFfYdH8Gipec8Eeeu0xXdbba9frFj0=OqFfea0dXdd9vqai=hGuQ8kuc9pgc9s8qqaq=dirpe0xb9q8qiLsFr0=vr0=vr0dc8meaabaqaciaacaGaaeqabaqabeGadaaakeaacqWGRbWAcqGH9aqpdaWcaaqaaiabdIeainaaBaaaleaacqWGbbqqaeqaaOGaeiikaGIaeGymaeJaeyOeI0IaemOzay2aaSbaaSqaaiabdgeabbqabaGccqGGPaqkaeaacqWGMbGzdaWgaaWcbaGaemyqaeeabeaakiabdIeainaaBaaaleaacqWGcbGqaeqaaaaaaaa@3C88@

Since 1 - *f*_*A *_is the true frequency of allele B, (we denote it *f*_*B*_), we can simplify this further:

k=HAHB⋅fBfA     (3)
 MathType@MTEF@5@5@+=feaafiart1ev1aaatCvAUfKttLearuWrP9MDH5MBPbIqV92AaeXatLxBI9gBaebbnrfifHhDYfgasaacH8akY=wiFfYdH8Gipec8Eeeu0xXdbba9frFj0=OqFfea0dXdd9vqai=hGuQ8kuc9pgc9s8qqaq=dirpe0xb9q8qiLsFr0=vr0=vr0dc8meaabaqaciaacaGaaeqabaqabeGadaaakeaacqWGRbWAcqGH9aqpdaWcaaqaaiabdIeainaaBaaaleaacqWGbbqqaeqaaaGcbaGaemisaG0aaSbaaSqaaiabdkeacbqabaaaaOGaeyyXIC9aaSaaaeaacqWGMbGzdaWgaaWcbaGaemOqaieabeaaaOqaaiabdAgaMnaaBaaaleaacqWGbbqqaeqaaaaakiaaxMaacaWLjaWaaeWaaeaacqaIZaWmaiaawIcacaGLPaaaaaa@3F20@

All values on the right-hand side of formula (3) are known, so we can estimate *k *in any pooling experiment for which we know the true allele frequencies without the need to observe heterozygous individuals. Indeed, for a heterozygous individual, the true ratio of the allele frequencies *f*_*B*_/*f*_*A *_of alleles A and B equals to one and therefore the formula above simplifies to k=HAHB
 MathType@MTEF@5@5@+=feaafiart1ev1aaatCvAUfKttLearuWrP9MDH5MBPbIqV92AaeXatLxBI9gBaebbnrfifHhDYfgasaacH8akY=wiFfYdH8Gipec8Eeeu0xXdbba9frFj0=OqFfea0dXdd9vqai=hGuQ8kuc9pgc9s8qqaq=dirpe0xb9q8qiLsFr0=vr0=vr0dc8meaabaqaciaacaGaaeqabaqabeGadaaakeaacqWGRbWAcqGH9aqpdaWcaaqaaiabdIeainaaBaaaleaacqWGbbqqaeqaaaGcbaGaemisaG0aaSbaaSqaaiabdkeacbqabaaaaaaa@33CD@, which equates with formula (1) – the standard way of estimating *k *from the observation of a heterozygous individual.

The situation in DNA pooling with Affymetrix arrays is only slightly different, as the RAS score is not just the ratio of the intensities for alleles A and B, but it is also corrected for the non-specific hybridisation (the mismatch intensity). The simplified formula of RAS is the median RAS of all quartets on one strand and approximates to:

RAS=(PMA−MM)(PMA−MM+PMB−MM)
 MathType@MTEF@5@5@+=feaafiart1ev1aaatCvAUfKttLearuWrP9MDH5MBPbIqV92AaeXatLxBI9gBaebbnrfifHhDYfgasaacH8akY=wiFfYdH8Gipec8Eeeu0xXdbba9frFj0=OqFfea0dXdd9vqai=hGuQ8kuc9pgc9s8qqaq=dirpe0xb9q8qiLsFr0=vr0=vr0dc8meaabaqaciaacaGaaeqabaqabeGadaaakeaacqWGsbGucqWGbbqqcqWGtbWucqGH9aqpdaWcaaqaaiabcIcaOiabdcfaqjabd2eannaaBaaaleaacqWGbbqqaeqaaOGaeyOeI0Iaemyta0Kaemyta0KaeiykaKcabaGaeiikaGIaemiuaaLaemyta00aaSbaaSqaaiabdgeabbqabaGccqGHsislcqWGnbqtcqWGnbqtcqGHRaWkcqWGqbaucqWGnbqtdaWgaaWcbaGaemOqaieabeaakiabgkHiTiabd2eanjabd2eanjabcMcaPaaaaaa@49B1@

where *PM*_*A *_is the intensity of the perfect match for allele A, *PM*_*B *_is the intensity of the perfect match for allele B and *MM *is the mean of the two mismatches [20]. The full algorithm keeps the RAS value bound between 0 and 1. Effectively the RAS score given by the software is the RAS of allele A. In order to use formula (3), we first express it in terms of intensities of allele A only, namely *f*_*B *_= 1 - *f*_*A *_and *H*_*B *_= 1 - *H*_*A*_, and therefore formula (3) can be re-written as

k=HA−HAfAfA−HAfA.
 MathType@MTEF@5@5@+=feaafiart1ev1aaatCvAUfKttLearuWrP9MDH5MBPbIqV92AaeXatLxBI9gBaebbnrfifHhDYfgasaacH8akY=wiFfYdH8Gipec8Eeeu0xXdbba9frFj0=OqFfea0dXdd9vqai=hGuQ8kuc9pgc9s8qqaq=dirpe0xb9q8qiLsFr0=vr0=vr0dc8meaabaqaciaacaGaaeqabaqabeGadaaakeaacqWGRbWAcqGH9aqpdaWcaaqaaiabdIeainaaBaaaleaacqWGbbqqaeqaaOGaeyOeI0IaemisaG0aaSbaaSqaaiabdgeabbqabaGccqWGMbGzdaWgaaWcbaGaemyqaeeabeaaaOqaaiabdAgaMnaaBaaaleaacqWGbbqqaeqaaOGaeyOeI0IaemisaG0aaSbaaSqaaiabdgeabbqabaGccqWGMbGzdaWgaaWcbaGaemyqaeeabeaaaaGccqGGUaGlaaa@40AF@

Now we can use the RAS score as the measure of the intensity of allele A (i.e. substitute *H*_*A *_by RAS):

k=RAS−RAS⋅fAfA−RAS⋅fA.
 MathType@MTEF@5@5@+=feaafiart1ev1aaatCvAUfKttLearuWrP9MDH5MBPbIqV92AaeXatLxBI9gBaebbnrfifHhDYfgasaacH8akY=wiFfYdH8Gipec8Eeeu0xXdbba9frFj0=OqFfea0dXdd9vqai=hGuQ8kuc9pgc9s8qqaq=dirpe0xb9q8qiLsFr0=vr0=vr0dc8meaabaqaciaacaGaaeqabaqabeGadaaakeaacqWGRbWAcqGH9aqpdaWcaaqaaiabbkfasjabbgeabjabbofatjabgkHiTiabbkfasjabbgeabjabbofatjabgwSixlabdAgaMnaaBaaaleaacqWGbbqqaeqaaaGcbaGaemOzay2aaSbaaSqaaiabdgeabbqabaGccqGHsislcqqGsbGucqqGbbqqcqqGtbWucqGHflY1cqWGMbGzdaWgaaWcbaGaemyqaeeabeaaaaGccqGGUaGlaaa@4858@

The resulting value for *k *is in fact the correct value of *k *for each SNP observed in our experiment and it does not require the observation of heterozygous individuals.

This procedure inevitably results in the correct estimation of parental allele frequencies but exaggerates the error in estimating allele frequency in the offspring pools as this contains the error from both samples. It does not however result in any bias in estimating the performance of the pooled assay in determining the difference in allele frequencies between pools. The accuracy of that prediction, when compared with the true difference is the main outcome in our study.

## Authors' contributions

GK and MCO'D conceived of and designed the study

GK performed most of the analysis, contributed to interpretation of the data, and took the lead in drafting the manuscript

IN performed the bioinformatics work, most importantly deriving the correct allele frequencies for all SNPs from the HapMap database

LG constructed the DNA pools

VM performed the data corrections

MJO and MCO'D contributed to interpretation of the data and to the writing of the manuscript.
